# Analgesic Therapy in Postherpetic Neuralgia: A Quantitative Systematic Review

**DOI:** 10.1371/journal.pmed.0020164

**Published:** 2005-07-26

**Authors:** Kathleen Hempenstall, Turo J Nurmikko, Robert W Johnson, Roger P A'Hern, Andrew S.C Rice

**Affiliations:** 1Royal Hampshire County Hospital, Winchester, United Kingdom,; **2**Department of Neurological Science, School of Medicine, University of Liverpool, United Kingdom,; **3**Bristol Royal Infirmary and University of Bristol, United Kingdom,; **4**Royal Marsden NHS Trust, London, United Kingdom,; **5**Department of Anaesthetics, Intensive Care and Pain Medicine, Imperial College London, United Kingdom; Harvard Medical SchoolUnited States of America

## Abstract

**Background:**

Postherpetic neuralgia (PHN) is a complication of acute herpes zoster, which is emerging as a preferred clinical trial model for chronic neuropathic pain. Although there are published meta-analyses of analgesic therapy in PHN, and neuropathic pain in general, the evidence base has been substantially enhanced by the recent publication of several major trials. Therefore, we have conducted a systematic review and meta-analysis for both efficacy and adverse events of analgesic therapy for PHN.

**Methods and Findings:**

We systematically searched databases (MEDLINE 1966–2004, EMBASE 1988–2004, CINAHL 1982–2002, and PubMed [29 October 2004]) for trials of PHN. We also searched references of retrieved studies and review articles for further trials. We included trials that examined adult patients with PHN of greater duration than 3 mo, that were blinded, randomised, and had at least one measure of pain outcome. Dichotomous pain outcome data were extracted for 50% decrease in baseline pain using a hierarchy of pain/pain-relief measurement tools. Where available, dichotomous data were also collected for adverse events. Calculated estimates of efficacy included relative benefit and number needed to treat.

Of 62 studies identified, 35 were randomised controlled trials. Of these, 31 were placebo controlled and suitable for meta-analysis, from which it was possible to extract dichotomous efficacy outcome data from 25.

This meta-analysis revealed that there is evidence to support the use of the following orally administered therapies: tricyclic antidepressants, “strong” opioids, gabapentin, tramadol, and pregabalin. Topical therapies associated with efficacy were lidocaine 5% patch and capsaicin. Finally, a single study of spinal intrathecal administration of lidocaine and methyl prednisolone demonstrated efficacy, although this has yet to be replicated.

Data suggest that the following therapies are not associated with efficacy in PHN: certain NMDA receptor antagonists (e.g., oral memantine, oral dextromethorphan, intravenous ketamine), codeine, ibuprofen, lorazepam, certain 5HT_1_ receptor agonists, and acyclovir. Topical administration of benzydamine, diclofenac/diethyl ether, and vincristine (iontophoresis) are similarly not associated with efficacy, nor are intrathecal administration of lidocaine alone or epidural administration of lidocaine and methylprednisolone, intravenous therapy with lidocaine, subcutaneous injection of Cronassial, or acupuncture. However, many of the trials that demonstrated a lack of efficacy represented comparatively low numbers of patient episodes or were single-dose studies, so it may be appropriate to regard such interventions as “not yet adequately tested” rather than demonstrating “no evidence of efficacy.” Topical aspirin/diethyl ether has not been adequately tested.

**Conclusion:**

The evidence base supports the oral use of tricyclic antidepressants, certain opioids, and gabapentinoids in PHN. Topical therapy with lidocaine patches and capsaicin is similarly supported. Intrathecal administration of methylprednisolone appears to be associated with high efficacy, but its safety requires further evaluation.

## Abstract 2

A systematic review of the evidence for treatment of postherpetic neuralgia summarises the therapies that seem to be most effective.

## Introduction

Postherpetic neuralgia (PHN) is a chronic neuropathic pain syndrome that may complicate recovery from an acute attack of herpes zoster. Despite advances in antiviral therapy during acute herpes zoster and the more recent introduction of vaccination against varicella zoster, PHN continues to be a significant clinical problem, with up to 25% of patients developing persistent neuropathic pain after acute herpes zoster reactivation [1]. Acute herpes zoster, and, consequently, PHN, particularly afflicts the immunocompromised and elderly, a fact that has serious implications for health-care delivery in the context of ageing populations in the developed world and the worldwide spread of HIV disease. Left untreated, PHN can become a severe and debilitating condition affecting all aspects of a patient's life [2].

The nature of neuropathic pain in PHN is variable; it may be described as continuous or paroxysmal, evoked or spontaneous, burning or lancinating, and be associated with a range of other sensory abnormalities in the skin [3]. This variability in symptomatology could imply that a variety of different pain mechanisms may be operating in different patients with PHN or in the same patient at different points in time [3,4]. This hypothesis has led to the suggestion that treatment plans could be optimised for individual patients on the basis of symptoms or even mechanisms [5]. However, the current situation is that the existing evidence base for therapies in PHN is constructed largely from clinical trials of analgesics that have examined PHN as a single disease entity. Furthermore, no substantial evidence base exists to relate specific sets of symptoms or signs to the efficacy of specific drugs, and no simple validated methods exist to determine which neuropathic pain mechanism(s) may be operating in a single patient. These factors dictate that current neuropathic pain treatment paradigms are forced to focus on PHN as a single disease entity.

No single treatment has been shown to be completely effective for all sufferers of PHN, and in the practical clinical scenario combinations of analgesic drugs are usually required to achieve partial relief of pain. Although there are an increasingly large number of trials that compare various analgesics to placebo, very few directly compare single therapies for which an evidence base exists, or address the issue of combining treatments [6].

Although published systematic reviews have collated the evidence base for analgesic therapy in PHN [7–9] and neuropathic pain in general [10], the evidence base has fundamentally and substantially altered since the publication of these reviews, with the appearance of several major studies [11–21], partly because of the increasing recognition of the usefulness of PHN as a clinical model of neuropathic pain for trials. Therefore, we have conducted a systematic review and meta-analysis of analgesic therapy for PHN, which includes these more recent trials.

## Methods

### Inclusion Criteria

The time at which the pain of zoster-associated pain becomes PHN is debated in the literature. We have used the definition of pain persisting for longer than 3 mo after the crusting of skin lesions following an acute attack of herpes zoster. Trials were sought that examined adult patients with zoster-associated pain for greater than 3 mo, were blinded, randomised, and had at least one clinically relevant measure of pain outcome. Unpublished, letter, and abstract-only studies were excluded as were studies on prevention of PHN and anecdotes. Studies where data for PHN were not analysed separately from other neuropathic pain syndromes were also excluded. All randomised controlled trials (RCTs) identified up to October 2004 were included.

### Identification of Studies

The following databases were searched without language restrictions: MEDLINE 1966–2004, EMBASE 1988–2004, CINAHL 1982–2002, and PubMed (29 October 2004). Search terms used were: “herpes zoster*,” “postherpetic neuralgia*,” “neuralgia*,” “pain*,” and “neuropathic*,” in combination with “randomised,” “random*,” “random allocation,” “double-blind,” “controlled clinical trials,” “trials,” and “study.” The Cochrane Controlled Trial Register and Cochrane Library (2004) database was searched using similar search terms. References of retrieved studies and review articles were also searched for further trials.

### Quality Assessment

Identified studies were independently assessed by at least two of four authors (KH, TJN, ASCR, and RWJ) in order to ascertain whether the inclusion criteria for PHN were met. The trials that met the PHN criteria were then quality scored by the same authors using the five point “Jadad” scoring system [22]. Studies were excluded if they achieved a score of less than three or if the enrolled study population was ten patients or less [23]. Disagreement between authors as to scores was adjudicated by ASCR to reach a consensus.

### Data Extraction

The following data were extracted from each study: drug or treatment examined, number of patients enrolled and analysed, study design and duration, dosing regimen, outcome measures used, pain-relief outcomes, minor and major adverse events, and withdrawals. For crossover studies, patient episodes were calculated as one episode representing the result for one patient completing one limb of the crossover, i.e., one patient who completed both active and placebo arms of a trial was counted as two patient episodes. For crossover studies, information was extracted regarding the provision of washout periods and also as to whether verification of the adequacy of any washout period was performed (e.g., return of pain intensity to baseline before second treatment period). Data were extracted by two of the authors (KH, TJN, or ASCR) independently. For the measurement of treatment efficacy, an outcome was considered clinically relevant if an improvement of 50% or greater in pain relief was achieved. Where possible, dichotomous data were extracted; when dichotomous data were not presented in the published study, the corresponding authors were contacted and asked to supply such data if they were still available. Due to the length of time that had elapsed since many of the publications, these data were often not available. Data were extracted for the longest follow-up period reported in each trial.

Because of the wide variety of outcome measures used across all of the reviewed papers, a hierarchy of outcome measures was implemented in line with the system used by several previous pain-treatment systematic reviews [8,23,24], as follows: 1) top two values on a five-point patient-reported global scale for pain relief or effectiveness or improvement; 2) top three values on a six-point patient-reported global scale for pain relief or effectiveness or improvement; 3) top value on a three-point patient-reported global scale for pain relief or effectiveness or improvement; 4) top two values on a four-point patient-reported categorical pain-relief scale; 5) 50% or greater reduction on a visual analogue or 11-point numerical rating scale for pain intensity.

Where available, dichotomous data were collected on adverse events. A major adverse effect was defined as an event that precipitated the withdrawal of the patient from the study, while an adverse event was considered minor if the patient continued the treatment and completed the trial. Some studies reported withdrawals not considered to be related to the treatment separately. However, this approach was not consistently the case across the included studies, so we adopted a conservative approach and included all withdrawals in the figures for major harm. Comparator trials using active controls were excluded from the safety analysis, in keeping with the system used by Collins et al. [8].

### Statistical Methods

A quantitative analysis was carried out on those trials where dichotomous data were available. Estimates of efficacy calculated were relative benefit (RB) and number needed to treat (NNT). The difference between active treatment and placebo was taken as statistically significant when the lower limit of the 95% confidence interval (CI 95%) for RB was greater than one. If tests of homogeneity were favourable, pooling of data was carried out for groups of similar treatments. A qualitative comment is made on those trials where no dichotomous data were obtainable.

Safety analysis was carried out on placebo-controlled trials using relative risk (RR) and number needed to harm (NNH). A fixed-effects model was used for calculating RB and RR with 95% CIs. A statistically significant improvement was noted when the lower limit of the 95% CI for RB was greater than one. Where RB or RR was not statistically significant, the method proposed by Altman was used to describe the CI for NNT or NNH [25]. Number needed to treat to benefit (NNTB) and number needed to treat to harm (NNTH) are given, so that if NNT was calculated as ten and the 95% CI 40 to −20, then this would be expressed as NNT10 (NNTH 20 to : to NNTB 40). If there were no responders in either the active or placebo-treated groups, then the RB was undefined and this situation was dealt with by the method proposed by Fleiss [26]. When calculating odds ratios, 0.5 was added to each of the cell frequencies in the 2 × 2 table showing response by treatment allocation. Although the absolute risks in the arms may vary between trials and may be affected by factors such as varying lengths of follow-up, the assumption was made that the RRs would be similar across trials that addressed the same question. Where possible, combined figures for classes of drugs were calculated based on RR estimates from each of the trials. Heterogeneity tests were performed on these trials; a value of *p* > 0.05 was interpreted to mean that it was appropriate to combine the RRs from different trials. Calculations were performed using the methods proposed by Armitage and Berry [27], programming being undertaken using Microsoft Excel 2000.

Some trials addressing a particular question will show treatment benefits that are clearly greater or less than the typical benefit observed in all the other trials addressing that question; in this case, there is heterogeneity between the trial outcomes (i.e., trials with a RR that differs from the overall RR more than would be expected by chance). The method proposed by Galbraith was used to identify such atypical trials [28]. If the treatment benefit is greater, we have described such trials as “high”; if it is less, we have described them as “low.”

This review is reported in accordance with the QUOROM guidelines [29].

## Results

**Figure 1:**
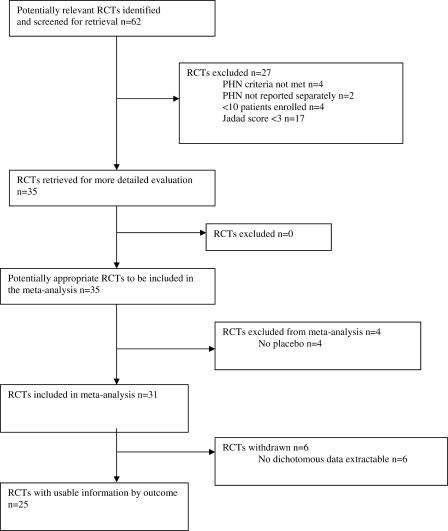
QUOROM Statement Flow Diagram

After the searches were completed, and obvious review articles, case and series studies, and anecdotes excluded, 62 articles were retrieved and independently reviewed by at least two of four of the authors (KH, TJN, RWJ, and ASCR). Twenty-seven studies were excluded at this stage (Figure 1). Details of excluded papers and, therefore, interventions that must be regarded as not having been adequately tested, are shown in Table 1.

**Table 1:**
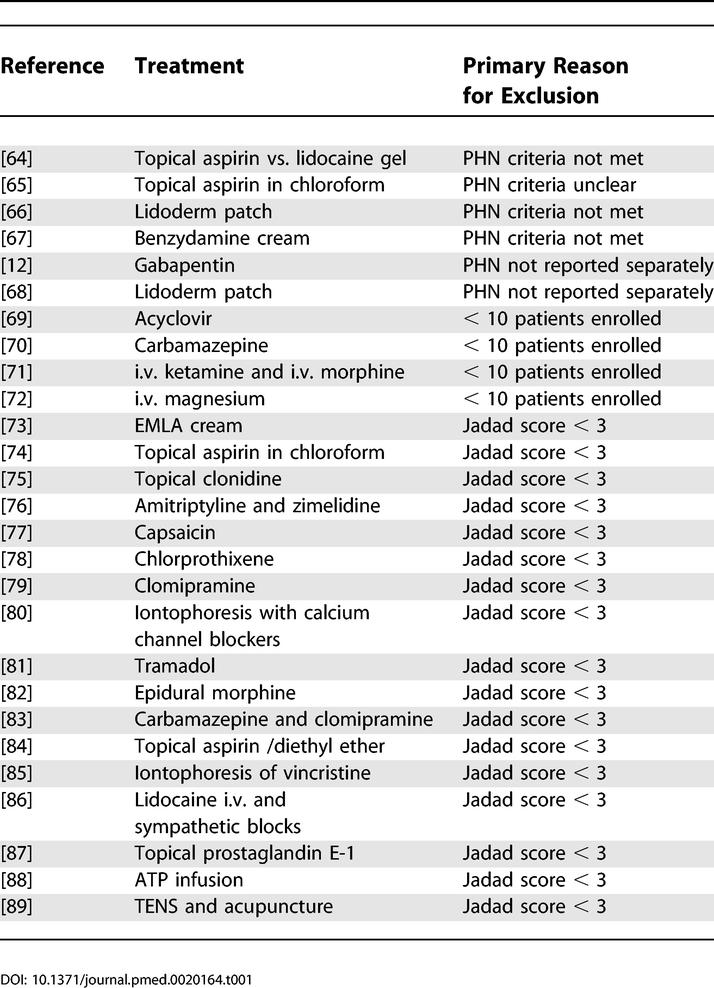
Studies Excluded from the Analysis and Therefore Intervention Not Adequately Tested

Of 35 trials retained for further analysis, 18 were of a crossover design and 17 were of a parallel group design (Table 2). Thirty-one trials were placebo controlled (including “active” placebo). Four trials were comparator studies without a placebo group and therefore could not be included in the meta-analysis. These trials compared amitriptyline to nortriptyline [30], amitriptyline to maprotiline [31], and intrathecal steroid to epidural steroid [17] and two doses of levorphanol [21]. Of the remaining 31 trials, we were able to extract dichotomous outcome data for efficacy meta-analysis from 25 (Table 3). Qualitative comment has been made on the included studies from which dichotomous data could not be extracted.

**Table 2:**
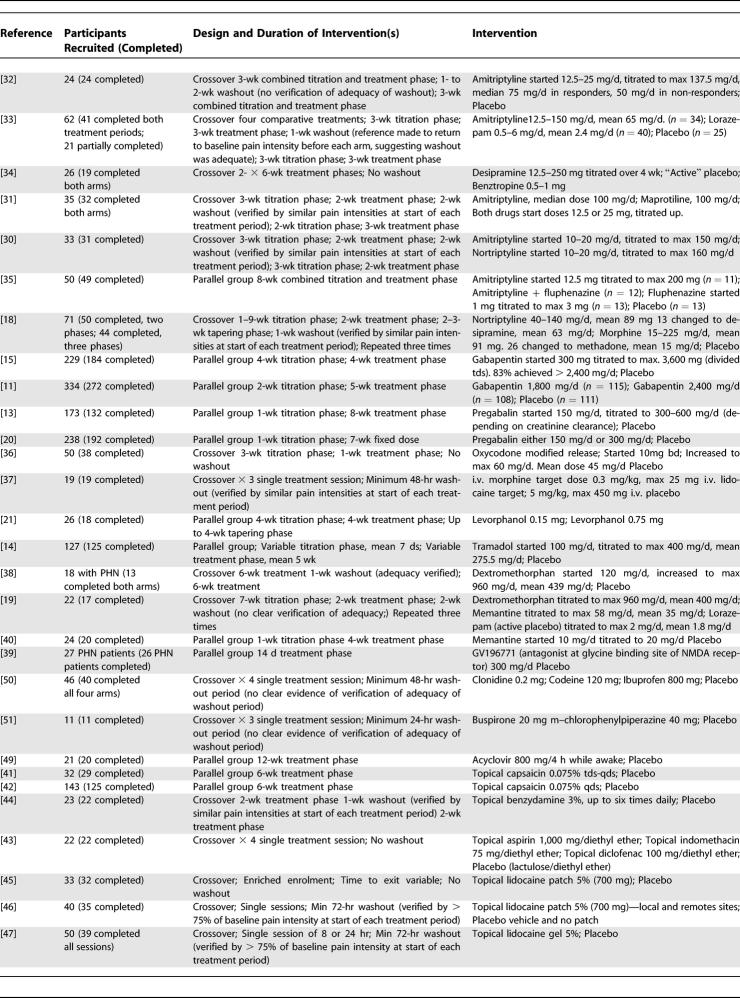
Studies Included in the Efficacy Analysis

**Table 2:**
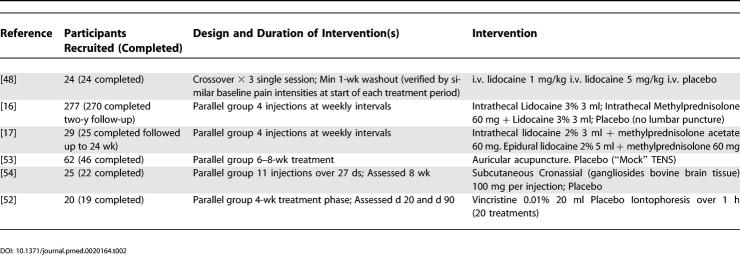
Continued

**Table 3:**
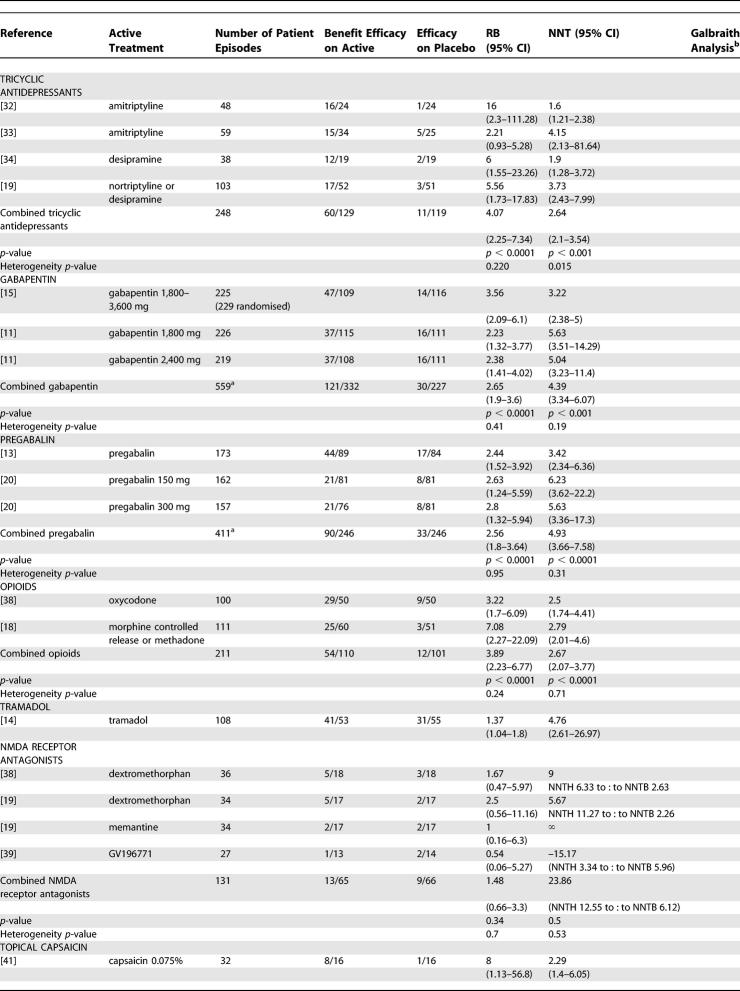
Summary of Data from Placebo-Controlled Trials for Which Dichotomous Data for Efficacy Could Be Extracted

**Table 3:**
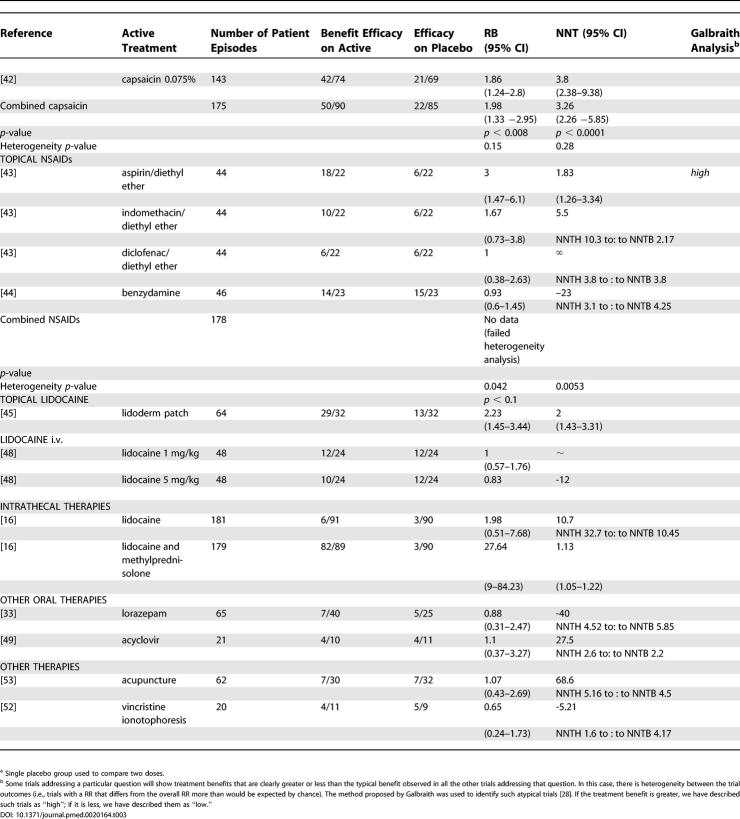
Continued

Data extracted were for the longest follow-up period reported in each trial. In the vast majority of trials, this was only until the end of the treatment period, with the exception of the intrathecal methylprednisolone studies [16,17] that reported follow-up periods to 24 wk and 2 y, and one study that examined amitriptyline and followed ten “good responders” for 2 y [31].

In 14 studies, we could not find any reference to intent-to-treat analysis. In these studies, the percentage of non-completers varied between 1% and 24%. In seven studies, all recruited patients completed the study, and an additional 13 studies specifically indicated that an intent-to-treat analysis had been performed.

Of the 31 included studies, 18 were a crossover design, notably those published longer ago (see Table 2). The design of 14 of these studies included a “washout” period, and ten of which included data that had the effect of verifying the adequacy of the washout period (e.g., return to baseline pain intensity before a treatment period).

### Efficacy of Antidepressants

Seven RCTs (297 participants recruited) investigated a range of tricyclic antidepressants, and six of these were of a crossover design. Five of these trials had extractable dichotomous outcome data, four of which compared amitriptyline, nortriptyline, or desipramine to placebo (Table 3) [18,32–34]. One trial used either nortriptyline or desipramine (the desipramine was used as a “back-up” medication when either the adverse effects of nortriptyline were intolerable or the dose could not be increased sufficiently). The reason for this design was that the primary aim of the study was to evaluate two classes of drug (opioid and tricyclic antidepressants) in general, rather than specific drugs [18]. These four trials accounted for 248 patient episodes and showed significant benefit associated with tricyclic antidepressant therapy, both individual trials and as pooled data (NNT 2.64 [2.1–3.54]) (Table 3). The fifth trial was a direct comparison of amitriptyline against maprotiline and therefore could not be included in the meta-analysis, but analysis of the data revealed that amitriptyline was associated with efficacy (50% pain reduction) in 47% (15/32) of patients and maprotiline in 38% (12/32); there was no significant difference between the treatments [31].

There were two trials where no dichotomous data were available. In one study, amitriptyline and nortriptyline were equally effective, with approximately 50% achieving a “good” response (top two values on five-point scale) [30]. A parallel design trial, with small numbers of patients in each arm, compared amitriptyline, fluphenazine, and a combination of the two to placebo [35]. Amitriptyline showed significant benefit; however, the addition of fluphenazine made no significant improvement. Fluphenazine alone was no better than placebo.

### Efficacy of Gabapentinoids

Two parallel group trials that examined the efficacy of gabapentin in PHN were included in the meta-analysis (559 patient episodes) [11,15]. In both trials, the dose of gabapentin was titrated over 1–2 wk. In one study, the target dose was 3,600 mg/d, a minimum of 1,200 mg/d was acceptable, and 65% achieved the target dose, while 83% received at least 2,400 mg/d [15]. The other trial compared the efficacy of two fixed doses of gabapentin, 1,800 mg/d and 2,400 mg/d [11]. Unlike the previous study, patients unable to attain the target doses were counted as withdrawals. Both doses showed efficacy with no significant difference between the two. The pooled results for gabapentin gave a NNT (50% pain reduction) of 4.39 (3.34–6.07) (Table 3).

We located two parallel group placebo-controlled trials in which the analgesic efficacy of pregabalin was investigated (411 patient episodes) [13,20], both trials revealing a superiority over placebo. In one trial, a pregabalin dose of either 300 or 600 mg/d, depending on creatinine clearance, was administered as an attempt to obtain a predicted pregabalin plasma concentration in all patients [13]. The pooled NTT for a 50% pain reduction with pregabalin was 4.93 (3.66–7.58) (Table 3).

### Efficacy of Opioids

We identified three RCTs that compared a conventional opioid to placebo, two crossover studies that investigated orally administered preparations [36], and one crossover trial with intravenous (i.v.) morphine [37]. A parallel group trial compared two doses of levorphanol [21]. Only the two crossover RCTs that examined orally administered medication had extractable dichotomous data (211 patient episodes), both of which demonstrated efficacy. Oxycodone, titrated up to a maximum of 60 mg/d [36], and controlled-release morphine (mean dose 91 mg/d) (or methadone [mean dose 15 mg/d] as a back-up medication if the morphine was not tolerated) [18] were associated with greater pain relief than placebo (Table 3). Pooled results for opioids yielded a NNT of 2.67 (2.07–3.77). The Raja et al. study also made a direct comparison of morphine/methadone to tricyclic antidepressants; the NNT for antidepressants was 3.73 (2.43–7.99) and for morphine/methadone 2.79 (2.01–4.6) [18].

In one non-placebo controlled parallel group study, two doses of levorphanol were compared [21]. Of the 26 PHN patients who entered the study, those treated with the 0.15-mg dose of levorphanol achieved a 14% reduction in baseline pain intensity, while the 0.75-mg dose was associated with a 33% reduction. When data from the 18 PHN patients who completed the study were analysed, this reduction was 10% and 42%, for the low- and high-dose groups, respectively.

The RCT that investigated i.v. morphine (0.3 mg/kg over 1 h, average dose 19.2 mg) assessed pain relief for 120 min, and morphine treatment was associated with a significant improvement in pain relief, with a VAS score of 44.9 ± 35.6 for morphine compared to 22.2 ± 32.8 for placebo [37].

We identified a single placebo-controlled parallel group RCT (108 patient episodes) that demonstrated the efficacy of orally administered controlled release tramadol (average titrated dose 275.5 mg/d), which yielded an NNT of 4.76 (2.61–26.97) [14] (Table 3). The wide 95% CI indicates that replication of this study is required before efficacy can be firmly stated.

### Efficacy of Drugs Acting at *N*-Methyl-D-Aspartate (NMDA) Glutamate Receptors

We were able to extract dichotomous data from three RCTs (131 patient episodes) that compared NMDA receptor antagonists to placebo, none of which demonstrated a superior efficacy over placebo (Table 3). Two studies were of a crossover design: one compared dextromethorphan to placebo (using benztropine as an active placebo) [38], and the other had three treatment arms using dextromethorphan, memantine, and lorazepam (as an active placebo) [19]. The third study was of a parallel group design in which GV196771 (300 mg/d), an antagonist at the glycine binding site of NMDA receptor, was found to have no superiority over placebo when the data for the primary or secondary pain outcomes measures were analysed, although it did have a reducing effect on the area of static and dynamic mechanical allodynia [39].

In one further parallel group study, memantine (20 mg/d) was shown not to be superior to placebo, although we were unable to extract dichotomous outcome data from this study [40].

### Efficacy of Topically Administered Treatments

Two parallel group studies (175 patient episodes) compared capsaicin cream (0.075% cream tds-qds) to placebo, analysis of which yielded a pooled NNT of 3.26 (2.26–5.85) (Table 3) [41,42]. The problems of maintaining adequate blinding to treatment in capsaicin studies, because of the sensations that this treatment evokes on application, are well documented. However, the choice of a parallel group design does somewhat mitigate against this deficiency.

Dichotomous data were extracted from two RCTs (178 patient episodes) that compared topical anti-inflammatory preparations to placebo. A crossover trial assessed single doses of aspirin (median dose 1,000 mg), indomethacin (median dose 75 mg), and diclofenac (median dose 100 mg) in diethyl-ether against placebo [43]. The aspirin and indomethacin preparations were associated with pain relief, while the diclofenac preparation was not (Table 3). However, significant heterogeneity was detected in these studies, and combined data could not be extracted.

A crossover trial of benzydamine 3% cream found no significant differences between active and placebo treatments (Table 3) [44].

Three RCTs (123 participants recruited) of topical lidocaine were identified; however, only one (64 patient episodes) had extractable dichotomous data for efficacy estimates (Table 3): An enriched enrolment crossover trial that included only participants who had reported moderate or greater pain relief from lidocaine patches in an open label study, compared lidocaine patch (5% lidocaine, 700 mg/patch, up to 3 patches/d) to placebo [45]. The primary outcome measure was “time to exit” from treatment; however, a secondary measure (highest level of pain relief sustained over at least 5 d) allowed an NNT of 2.00 (1.43–3.31) to be calculated (Table 3). In another study, lidocaine 5% patches were compared to two placebos (vehicle and “no patch”) in a single 12-h session; the authors reported that the lidocaine patch was associated with higher pain-relief scores compared to either placebo treatment [46]. In another RCT, 5% lidocaine gel was applied to painful areas of skin for single sessions of 24 h under an occlusive dressing or 8 h with no dressing [47]. The efficacy of lidocaine gel was compared to both vehicle placebo and application of lidocaine gel to a site remote from the area of PHN, while locally applied lidocaine gel was associated with analgesia efficacy compared to placebo. There was no significant difference between placebo and remote site treatments.

### Efficacy of i.v. Lidocaine

Although not practical for long-term therapy of PHN, treatment with i.v. lidocaine has been tested in two RCTs, and we were able to extract dichotomous outcome data from one of these: Neither lidocaine 1 mg/kg (48 patient episodes) nor 5 mg/kg (48 patient episodes), infused over 2 h were associated with superior pain relief than saline infusion in a crossover study (Table 3) [48]. The second trial was also of a crossover design and compared placebo, morphine 0.3 mg/kg, and lidocaine 5 mg/kg infused for 1 h (average total dose of lidocaine 316 mg) and revealed no significant difference between lidocaine and placebo [37].

### Efficacy of Intrathecally (i.t.) and Epidurally Administered Drugs

A parallel group study (total of 270 patient episodes) was identified that compared 3% lidocaine (3 ml, i.t.) (91 patient episodes), 3% lidocaine (3 ml, i.t.), plus 60 mg methylprednisolone (i.t.) (89 patient episodes) and no treatment (90 patient episodes) [16]. Intrathecal injections were carried out weekly for 4 wk and patients followed up for 2 y. Analysis of extracted dichotomous data revealed efficacy associated with the lidocaine/methylprednisolone treatment (NNT 1.13 [1.05–1.22]), but not with i.t. lidocaine alone (Table 3).

Another parallel group study (25 patient episodes) that compared a mixture of lidocaine 2% (3 ml) plus 60 mg of methylprednisolone administered i.t. with lidocaine 2% (5 ml) plus 60 mg of methylprednisolone administered epidurally was identified [17]. The lack of a placebo group dictated that this study could not be included in the meta-analysis, but the data revealed that the intrathecal treatment was associated with efficacy (50% pain reduction) in 92% (12/13) of patients whilst efficacy was seen in 17% (2/12) of patients who received the epidural treatment. If the epidural treatment is assumed to be a placebo, an NNT for the intrathecal therapy could be calculated at 1.32 (0.99–2.00).

### Efficacy of Miscellaneous Treatments

Two RCTs examined the benzodiazepine lorazepam: in one crossover study (65 patient episodes), lorazepam (0.5–6 mg/d) was not shown to be superior to placebo (Table 3) [33]. Lorazepam was also used as an active placebo in a crossover study (34 patient episodes) in which two NMDA receptor antagonists and lorazepam (mean dose 1.8 mg/d) were compared; no significant pain relief was reported between the groups (Table 3) [19].

We were able to extract dichotomous data from a parallel group study (21 patient episodes) that compared acyclovir (800 mg/4 h for 12 wk) to placebo, in which no benefit was attributable to acyclovir (Table 3) [49].

We were unable to extract dichotomous efficacy data from a further two crossover studies: one multi-arm study (160 patient episodes) compared single doses of codeine 120 mg, ibuprofen 800 mg, clonidine 0.2 mg to placebo, and there was no significant difference between treatments, but clonidine had some efficacy when compared to placebo [50]. In the second trial (33 patient episodes), the 5HT_1_ agonists buspirone and M-chloropiperazine were found to have no benefit when compared to placebo [51].

Transdermal iontophoresis of 0.01% (20 ml) vincristine showed no benefit for pain relief in a parallel group study (20 patient episodes) [52].

We were able to extract dichotomous data from one parallel group study (62 patient episodes) that compared auricular or body acupuncture to “mock TENS” as placebo; no analgesic benefit was associated with acupuncture (Table 3) [53].

We were unable to extract dichotomous outcome data for efficacy from a parallel group RCT (25 participants recruited, 22 completed) that compared subcutaneous injection of a mixture of bovine gangliosides (Cronassial) to placebo; no superiority over placebo was demonstrated [54].

### Adverse Events

Of the trials from which we were able to extract dichotomous data for efficacy, we were able to extract dichotomous data for minor and major harm from 18 and 20 reports, respectively (Tables 4 and 5).

**Table 4:**
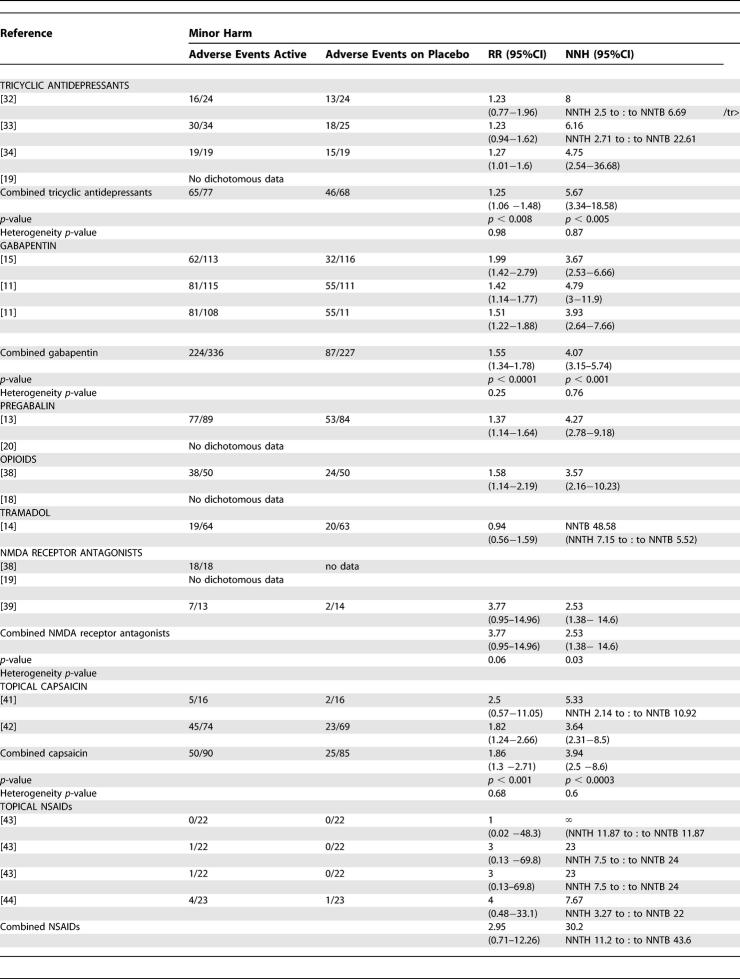
Summary of Data from Placebo-Controlled Trials for Which Dichotomous Data for Minor Harm Could Be Extracted

**Table 4:**
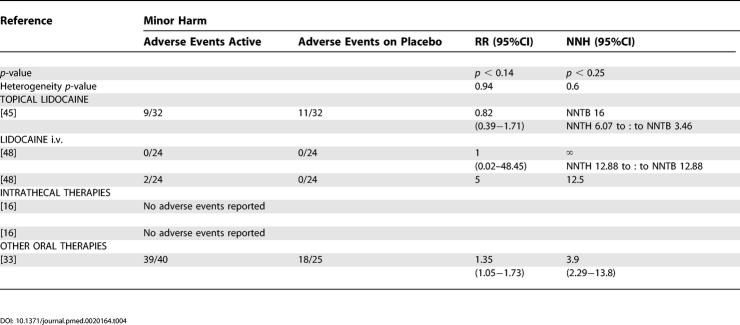
Continued

**Table 5:**
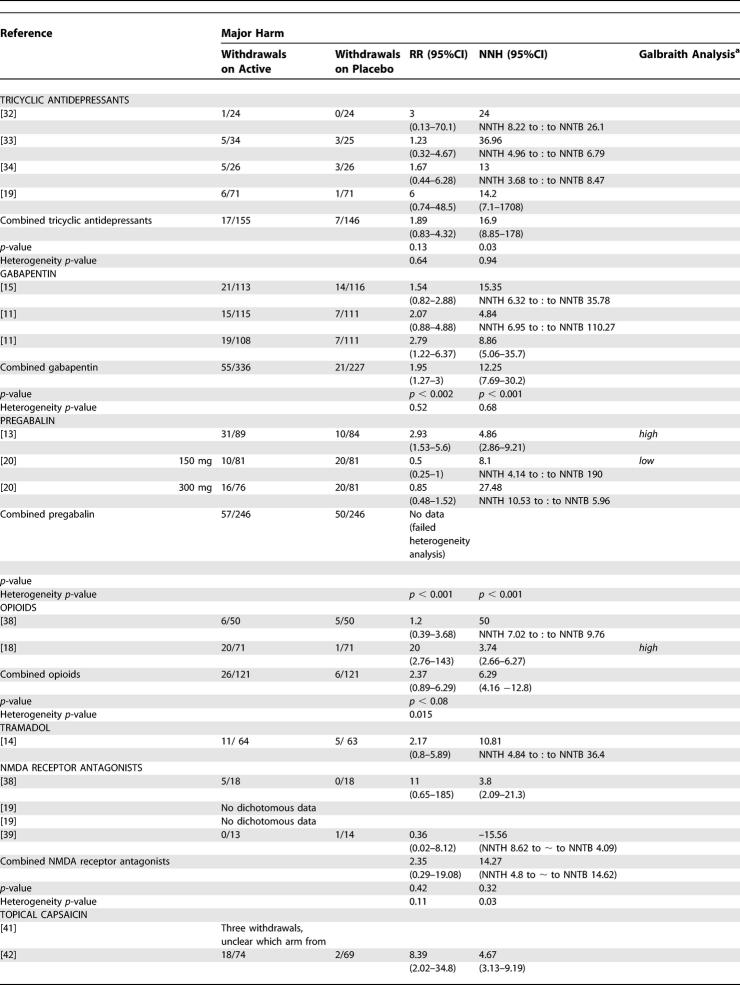
Summary of Data from Placebo-Controlled Trials for Which Dichotomous Data for Major Harm Could Be Extracted

**Table 5:**
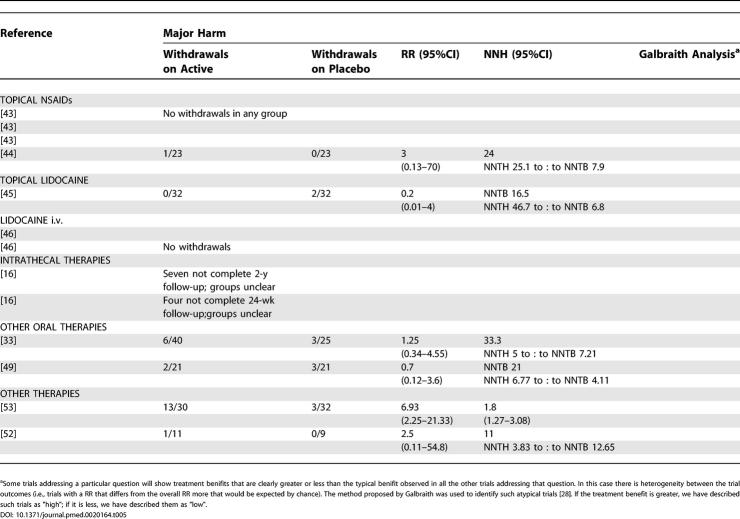
Continued

For tricyclic antidepressants, the pooled analysis revealed that 84% of participants reported minor adverse events (NNH 5.67 [3.34—18.58]) (Table 4). In addition to dizziness and sedation, the most frequently reported were anticholinergic in nature (dry mouth, constipation). However, the incidence of these varied between studies (e.g., 13–74% incidence of dry mouth) [18,34]. Three trials noted that adverse events were more frequent during the titration phase and then reduced during the maintenance phase [18,34]. Major adverse events: withdrawals were relatively small in number and again related mostly to sedation and other anticholinergic effects, one patient treated with desipramine developed left bundle branch block, and one treated with amitriptyline erythema multiforme [32,34]. The combined NNH for major adverse events was 16.9 (8.85–178) (Table 5).

The pooled results for gabapentin gave a NNH of 4.07 (3.15–5.74) for minor harm and 12.25 (7.69–30.2) for major harm (Tables 4 and 5). The most frequently reported adverse events were dizziness and somnolence. One death was reported in each trial—both considered to be unrelated to study medication—one in a gabapentin-treated patient and one in a placebo-treated patient. Withdrawals in the first pregabalin trial were mostly related adverse events (28 out of 31 withdrawals in the active treatment group), giving a NNH of 4.27 (2.78–9.18) and 4.86 (2.86–9.21) for minor and major harm, respectively (Tables 4 and 5) [13]. Compared to the gabapentin trials, the titration period was more rapid, over 1 wk compared to 16 d and 4 wk. In the second pregabalin trial, an 83% incidence of minor adverse events was reported in the treatment group, but the incidence of withdrawal was highest in the placebo group [20]. Since there was significant heterogeneity between the two studies, the NNH data for pregabalin were not combined. The most frequent adverse events associated with pregabalin were dizziness and somnolence.

For both oxycodone and controlled-release morphine, constipation, nausea, and sedation were reported more often in the study medication groups than with placebo [36]. It was noted that nausea and sedation became less troublesome with time, but not constipation. Compared to tricyclic antidepressants, morphine was associated with a higher incidence of adverse events, in 72% compared to 36% of treated patients. The incidence of withdrawals differed between trials, with no significant difference between oxycodone and placebo, but a significant difference between morphine or methadone and placebo. The combined NNH for major harm was 6.29 (4.16–12.8); due to the detection of significant heterogeneity, we were unable to calculate a combined NNH for minor harm, but from one study [36], this was 3.57 (2.16–10.23) (Table 5). For tramadol, the NNT for major harm was calculated at 10.81 (Table 5).

For NMDA receptor antagonists, dichotomous data were available for minor adverse events from one trial 2.53 (1.38–14.6) [39] and for major adverse events from two trials [38,39] combined NNH 14.27 (NNTH of 4.8 to approximately NNTB of 14.62).

No systemic adverse events were reported in the capsaicin trials, but local irritation at the site of application of capsaicin was the most frequently reported local adverse event [41,42]. It was noted in both studies that these symptoms decreased or disappeared during the course of the trials. From the pooled data, a NNH for minor harm of 3.94 (2.5–8.6) was calculated (see Table 4). The most frequently reported reason for withdrawal was burning pain at the site of application. Dichotomous major adverse-events data were only extractable from one RCT that yielded an NNH of 4.67 (3.13–9.19) (Table 5) [42].

Lidocaine patches [45] were reported as being well tolerated with no systemic adverse events and only minor local skin reactions noted. There was no significant difference between lidocaine patch and placebo for minor adverse events. There were only three withdrawals, two in the placebo group associated with a skin reaction and worsening pain and one in the lidocaine-treated group with severe depression. For lidocaine and placebo gels, 38% of participants reported local skin reactions and 19% reacted to the Tegaderm dressing; there was no statistical difference between lidocaine and placebo [47].

The topical anti-inflammatory treatments were well tolerated, with a low incidence of local skin irritation (see Table 4) [43,44].

Whilst we were unable to extract dichotomous adverse-events data, no minor adverse effects were reported in either epidural or intrathecal lidocaine and methylprednisolone treated patients. Serial MRI scans were unchanged in all groups, and seven patients failed to complete the 2-y follow-up period, although no reasons were given for these withdrawals [16]. One patient who under went epidural injections had a cerebral haemorrhage 21 wk after injections, thought to be unrelated to treatment [17].

## Discussion

This systematic review of the literature suggests that there is evidence of analgesic efficacy (i.e., NNT < 5.00) in established PHN for the following orally administered therapies: tricyclic antidepressants, opioids, gabapentin, tramadol, and pregabalin (Table 3). There is also evidence that some topically administered therapies are associated with analgesic efficacy in appropriate patients: lidocaine patch and capsaicin. Intrathecal administration of lidocaine and methyl prednisolone is associated with long-lasting analgesia. However, an important proviso must be made when interpreting data derived using a meta-analytic approach; the evidence supporting some therapies have been derived from several studies, compromising a relatively large number of patient episodes (e.g., tricyclic antidepressants, gabapentinoids, and opioids), whereas others have been derived from single studies (e.g., tramadol or intrathecal methylprednisolone) and/or only a relatively low number of patient episodes (e.g., topical lidocaine and capsaicin). The data extracted from small and/or single unreplicated studies needs to be viewed with a particular degree of caution (see Tables 2 and 3).

Heterogeneity in clinical trial design and size is a frequent Achilles' heel of a meta-analytic approach. The European Medicines Agency has recently published guidelines for neuropathic pain studies, which hopefully, at least for regulatory studies conducted in Europe, will dictate that future trial methods will be more homogenous and, therefore, the data more comparable [55].

Some evidence supports the use of anticonvulsant (anti-epileptic) drugs other than gabapentinoids in neuropathic pain conditions other than PHN [10], but we could find no suitable trials that examined their use specifically in PHN. Furthermore, we consider the classification of drugs as “anticonvulsants” somewhat meaningless in this context, since these drugs have disparate mechanisms of action.

Compliance of patients with treatment is an important factor in the clinical effectiveness of therapies, and a major factor governing compliance is withdrawal due to side effects (major harm). However, the dichotomous data for adverse events that we have been able to extract must be viewed with some caution for a number of reasons. Firstly, we were unable to extract dichotomous adverse-events data for all studies from which we were able to extract efficacy data. Secondly, the methods for collecting adverse-event data varied widely between RCTs, with some trials actively seeking adverse-event details with specific scoring systems, while others relied only on patients volunteering such information. Thirdly, as with the efficacy data, many of the trials examined only short treatment periods—sometimes only single sessions—and also examined only a relatively small population of patients, a combination of factors that seriously reduces the ability of these RCTs to detect even relatively frequent adverse events. We elected to use rates for total adverse events, as many studies did not distinguish between events related or unrelated to therapy; however, the use of total adverse events as a measure could potentially yield a more global estimate of the pattern of adverse events and remove a subjective decision regarding such an association. Despite an appreciable frequency of minor adverse events, serious events were rare; the few deaths that were reported were considered to be unrelated to study medication (Tables 4 and 5).

Some data suggest that the following therapies are not associated with efficacy in PHN, within the dose range examined: oral administration of certain NMDA receptor antagonists (memantine, GV196771, and dextromethorphan), codeine, ibuprofen, lorazepam, 5HT_1_ receptor agonists, and acyclovir. Furthermore, topical administration of benzydamine, diclofenac/diethyl ether, and vincristine (iontophoresis) are not efficacious. Intrathecal administration of lidocaine or epidural administration of lidocaine and methylprednisolone are not associated with analgesia, nor is i.v. therapy with lidocaine or subcutaneous injection of Cronassial. Acupuncture is not associated with analgesic efficacy in PHN. However, it should be pointed out that, in contrast to the “positive” trials that contain large numbers of patient episodes, many of the trials where an effect was not demonstrated represented comparatively low numbers of patient episodes or were single-dose studies, so it may be appropriate to regard such interventions as “not yet adequately tested” rather than demonstrating “no evidence of efficacy.” Furthermore, the potential confounds of variations in pharmacokinetics and assessment of only a limited dose range/regimen dictate that a “lack of efficacy” statement cannot be made with complete confidence for these interventions.

In 16 publications, cutaneous allodynia was measured either qualitatively or quantitatively. Only bedside methods were used for mechanical (15 papers) and thermal allodynia (two papers). Quantitative data were presented from seven studies; the rest were purely qualitative and usually accompanied by brief comments only. In general, improvement in allodynia, or lack of it, paralleled changes in global pain scores. Tricyclic antidepressants, oxycodone, i.v. and topical lidocaine, and i.t. methylprednisolone reduced the intensity and/or area of allodynia, whereas conventional NMDA receptor antagonists did not, although there was an effect on the area of dynamic and static mechanical allodynia associated with the glycine site antagonist GV196771 [39]. No useful data in this respect were available for gabapentin, pregabalin, or tramadol.

The percentage of pain reduction or improvement that is clinically meaningful, and can therefore be employed as a dichotomous outcome measure, is controversial: the majority of systematic reviews published to date have reported dichotomous outcome data for efficacy in terms of 50% pain relief or reduction in baseline intensity. However, this measure, as far as we are aware, has been validated for acute pain only [56] and not for chronic neuropathic pain. Farrar et al. have validated the use of a 30% reduction in pain intensity as a clinically important dichotomous outcome measure for chronic pain [57]. We therefore attempted to obtain such 30% dichotomous outcome data for comparative purposes but were able to do so for only five studies; we have not reported such data since we consider that this would not reasonably reflect the breadth of included studies. However, we suggest that in future, RCTs in PHN should report responder rates for both 30% and 50% pain reduction, although of course it could be argued that a debate concerning the precise threshold for defining a responder is somewhat sterile, since it might be expected that the actual ratio of placebo to active treatment responders might be similar no matter which threshold was used, since the same threshold would be applied to both interventions.

In many studies, evaluation of overall pain was measured using the McGill Pain Questionnaire, but rarely were any additional useful data presented. Triyclic antidepressants, oxycodone, and i.t. methylprednisolone appeared to reduce brief pain paroxysms as well.

Mood was assessed using validated tools (BDI, POMS, HADS) in nine studies and quality of life (mainly SF36) in six. Simple linear or categorical scales were used in ten studies for sleep interference. In general, quality of life and sleep changed in parallel with improved pain scores, whereas changes in mood were inconsistent, the latter possibly due to methodological differences.

Whilst the development of clinical management guidelines for PHN, which are based upon high-quality evidence, is a highly desirable goal, such guidelines can of necessity include therapies only where evidence satisfying current criteria exists. Justification for a number of therapies in current use by some practitioners is based upon clinical experience and anecdote combined with research standards of yesteryear, and therefore such therapies have not been evaluated here. Unfortunately, since many of these therapies are of little current commercial interest to the pharmaceutical industry and do not fall within areas of interest of the major research funding organisations, it is extremely unlikely that an evidence base supporting or refuting such therapies will ever be compiled. Other considerations also influence evidence gathering relating to novel treatments: where regulatory approval requires evidence of efficacy and safety compared with placebo, it may not be commercially advantageous to undertake head-to-head studies directly comparing efficacy and safety against a current standard treatment and thus such data are sparse. Furthermore, many PHN patients take more than one therapy, and there is only a very limited evidence base of the additive and synergistic effects of combing therapies.

Nevertheless, from available evidence it is possible to produce guidelines for management, but only with the caveat that some therapies are excluded as they are not supported by high-quality evidence. Lack of evidence may be because adequate trials have shown no benefit or because no adequate trial has been undertaken.

A further problem arises from the fact that there is no single pathophysiology that defines the generation and persistence of PHN [3]. Future studies should use quantitative sensory evaluation to clearly categorise subsets of patients contained within a study population and be powered to permit separate evaluation of the data obtained for such subsets.

It is important to include economic considerations in development of a clinical management strategy, although it is recognised that this will vary between health-care systems. For example, where efficacy and adverse events of two drugs are similar, it would seem prudent to initiate treatment with the less expensive drug. For example, a treatment plan for oral therapy that proposes initial use of a tricyclic antidepressant, except where contra-indicated, might reserve the use of the more expensive gabapentinoids for patients, where the tricyclic antidepressant either fails to provide efficacy and/or is associated with unacceptable adverse events. Similarly, the efficacy of various opioids and tramadol supports their use, but perhaps as a second-line therapy in accordance with recommendations for the use of such drugs in chronic non-cancer pain [58].

With the above provisos, and using the evidence evaluation of this systematic review, the evidence base would support the first-line use of a tricyclic antidepressant for orally administered treatment of PHN, reserving the gabapentinoids for second-line use. However, a secondary proviso here might be a consideration of the wisdom of using, as first-line therapy, a group of drugs that have regulatory approval in many countries for the treatment of PHN (e.g., gabapentinoids) against a group that generally do not (e.g., tricyclic antidepressants). On the efficacy evidence, “strong” opioids could be considered as first- or second-line therapy, although the guidelines for use of opioids in non-malignant pain would suggest that opioids might be sensibly reserved for use following inadequate benefit from tricyclic antidepressants or gabapentinoids [58]. Logically, topical treatments might be considered to have a lower potential for generating systemic adverse effects than systemically administered therapies; therefore, the early use of topical lidocaine or capsaicin should be considered as a first line, especially where quantitative sensory evaluation (possibly supported by measurement of epidermal neuronal density in a skin biopsy [59]) has indicated that the patient falls into the “sensitised nociceptor” as opposed to “deafferentation” sub-group of PHN patients [3].

Intrathecal steroids appear to be associated with remarkable benefit in PHN patients [16], but this therapy may be potentially hazardous [60–62], and this trial has not yet been replicated. Therefore, we believe that a further high-quality RCT of this therapy is desirable before recommendations can be made regarding its use for PHN. This therapy is, of course, not suitable for PHN within the cranial nerves innervation territory.

There is little evidence regarding possible synergistic effects of the various treatments to support or refute the concomitant use of combinations of, e.g., tricyclic antidepressants, opioids, and gabapentinoids. However, it certainly seems logical that concomitant use of drugs with different mechanisms of action may offer additional benefit to PHN patients.

Although there is little direct evidence to support it, we would like to make a final recommendation that any treatment plan should recognise the importance of the biopsychosocial model of chronic pain and thus that any pharmacologically based management of PHN should be combined with advice on and management of psychological and social aspects [63].

**Patient Summary**

**Background**

Postherpetic neuralgia (PHN) is the pain that people sometimes get after shingles. It can be severe. Although many treatments have been tried for it, doctors do not agree on how to best treat it.

**What Did the Researchers Do?**

They looked systematically to find all the trials that have investigated treatments for PHN. They assessed each trial to see if it could provide useful results—e.g., if it was well designed, clear that they were treating patients with PHN, and that clear results could be taken from the trials. They also looked to see if the trials had assessed the possibility that the treatments could cause harm.

They found that there were a wide range of treatments that had been tried. The most reliable oral treatments were tricyclic anti-depressants, morphine-like drugs (opioids), and gabapentin (and related drugs). Some topical treatments also worked: e.g., a local anaesthetic lidocaine and a cream made from the active ingredient of chilli peppers—capsaicin. However, for many treatments there was not enough evidence to assess if they worked.

**What Do These Results Mean?**

This type of review is the most reliable form of evidence that doctors have available to them in deciding on treatment. Even so, the results are not conclusive. Future trials should be designed rationally to fill in the gaps of knowledge about the possible treatments for this disorder. In the meantime, however, there are some drugs that seem to work relatively well, and, outside of a clinical trial, these drugs should be used first.

**Where Can I Get More Information?**

Medline Plus discusses shingles and neuralgia more widely:

http://www.nlm.nih.gov/medlineplus/ency/article/000858.htm

The Neuropathy Trust has a Web site with patient information:

http://www.neurocentre.com

The Varicella Zoster Research Foundation Web site also has useful information for patients:

http://www.vzvfoundation.org

## Abbreviations

CI: confidence interval
i.t.: intrathecally
i.v.: intravenous
NMDA: N-methyl-D-aspartate
NNH: number needed to harm
NNT: number needed to treat
NNTB: number needed to treat to benefit
NNTH: number needed to treat to harm
PHN: postherpetic neuralgia
RB: relative benefit
RCT: randomised controlled trial
RR: relative risk
